# Supplementary material for: In-depth organic mass cytometry reveals differential contents of 3-hydroxybutanoic acid at the single-cell level
Source: Nat Commun. 2024 May 23;15:4387. doi: 10.1038/s41467-024-48865-2 (PMC11116506; doi:10.1038/s41467-024-48865-2)
Supplement: Supplementary file 1 — Supplementary Information [file 41467_2024_48865_MOESM1_ESM.pdf]

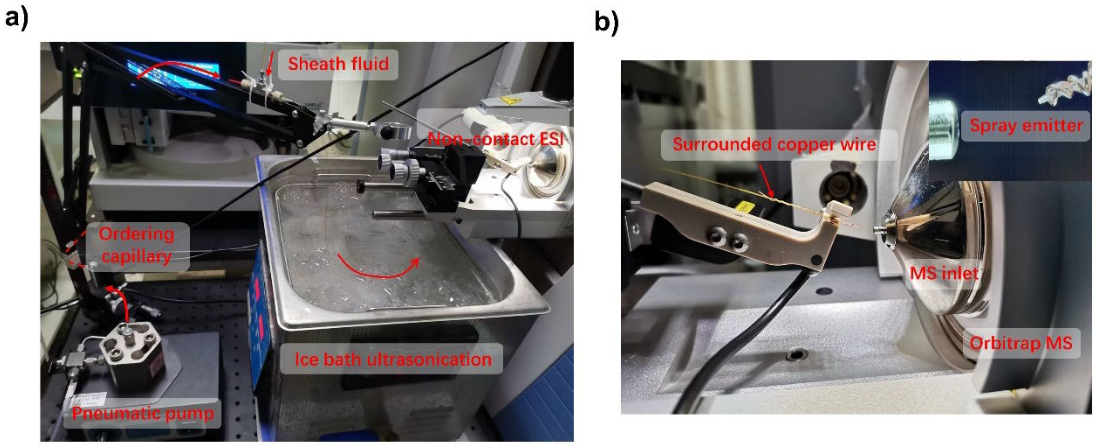

Supplementary Figure 1. Configuration of In-depth organic mass cytometry (ID-organic cytoMS). (a) Photo of ID-organic cytoMS showing its basic constructions: pneumatic pump for cell injection, ordering capillary, sheath liquid, ice bath ultrasonication, electrospray emitter, and high-resolution mass spectrometer. Cell suspension was pumped into ordering capillary to achieve further mono-dispersion. After contact with sheath liquid, ice bath sonication was utilized for rapid cell online lysis. Segmented lysate derived from single cell was subsequently analyzed by non-contact ESI-MS. (b) Configuration of non-contact ESI-MS. Spray emitter was surrounded by copper wire at the tip to generate spray electric field.

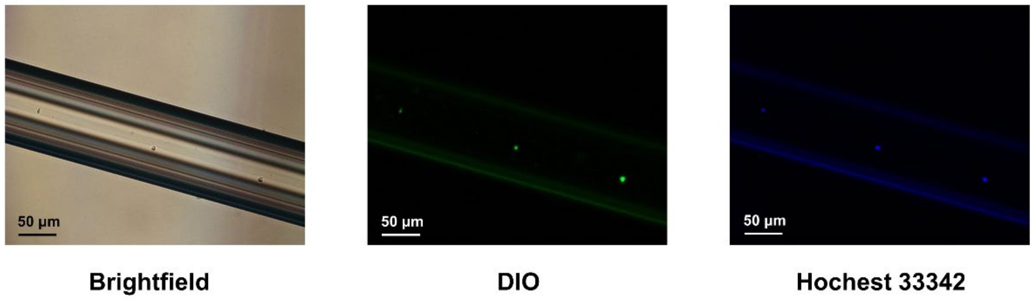

Supplementary Figure 2. Bright and florescence images of mono-dispersed MCF-7 cells in ordering capillary. (DIO stained for cell membrane and Hochest33342 stained for cell nuclei, scale bar: 50 μm)

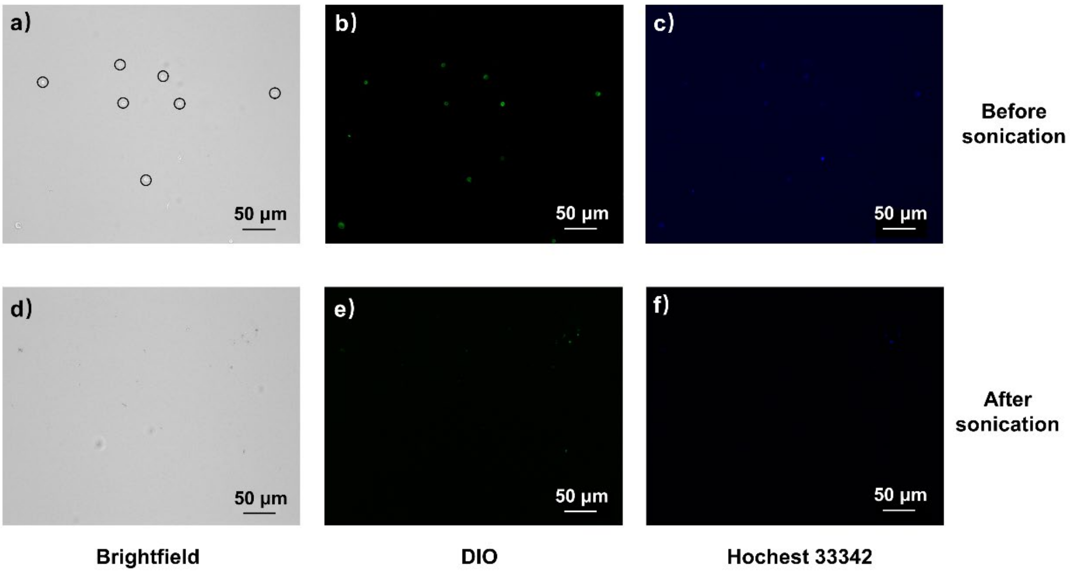

Supplementary Figure 3. Bright and florescence image of MCF-7 cells before and after sonication in the sheath liquid. Cellular structures were significantly destroyed upon sonication. (a) bright image before sonication; (b) stained with DIO before sonication (excitation wavelength: 488 nm); (c) stained with hochest 33342 before sonication (excitation wavelength: 405 nm); (d) bright image after sonication; (e) stained with DIO after sonication; (f) stained with hochest 33342 after sonication. scale bar: 50 μm

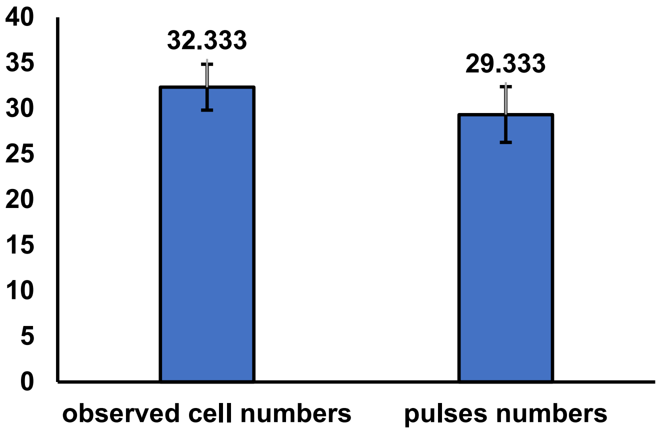

Supplementary Figure 4. Bar plot of overserved numbers and pulse numbers during 20 min. Cell suspension density was ~7000 cells /mL, N_2 pressure was set as 20 psi. Boxplot shows mean, Error bar represents the standard deviation

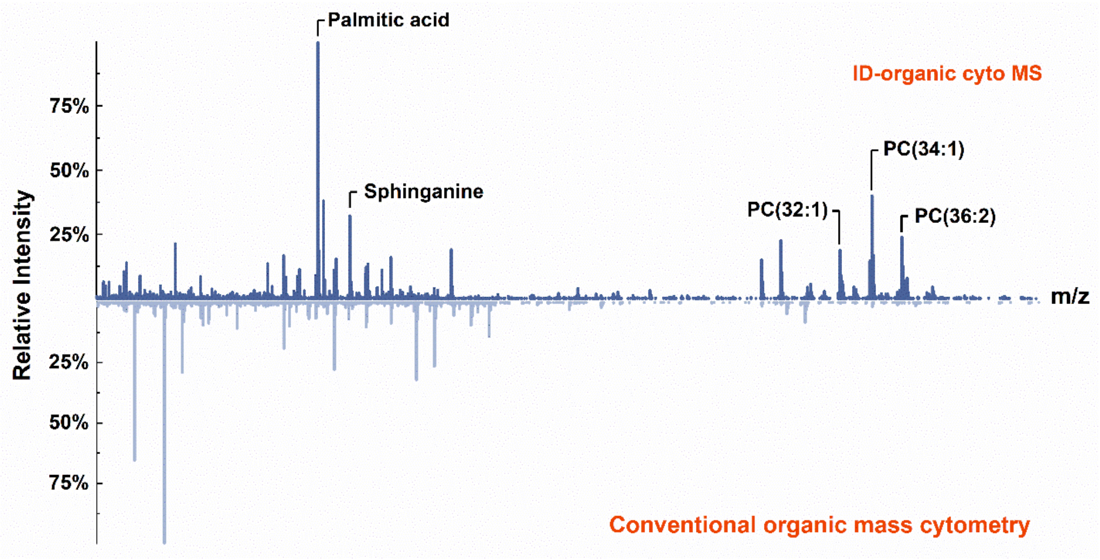

Supplementary Figure 5. Representative single-cell MS spectrum comparison between organic mass cytometry and ID-organic cytoMS in positive mode (mass range: 80-900).

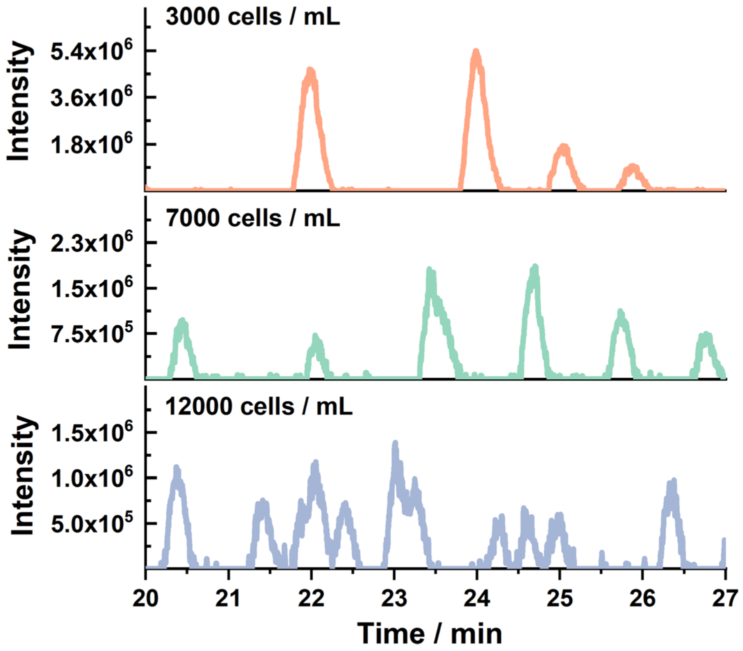

Supplementary Figure 6. Cell suspension density optimization. EIC of PC (34:1) in ID-organic cytoMS with cell density of ~3000 cells /mL, ~7000 cells /mL and ~12000 cells. Cell suspension with density of ~7000 cells /mL was optimal.

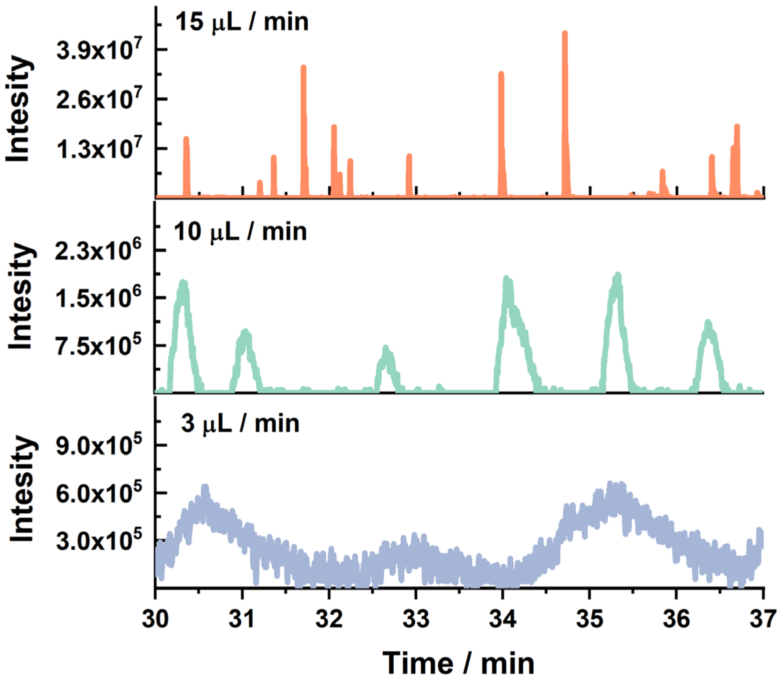

Supplementary Figure 7. Sheath liquid flow rate optimization. EIC of PC (34:1) in ID-organic cytoMS with sheath liquid flow rate of 15 μl/min, 10 μl/min and 3 μl/min. Flow rate of 10 μl/min was optimal.

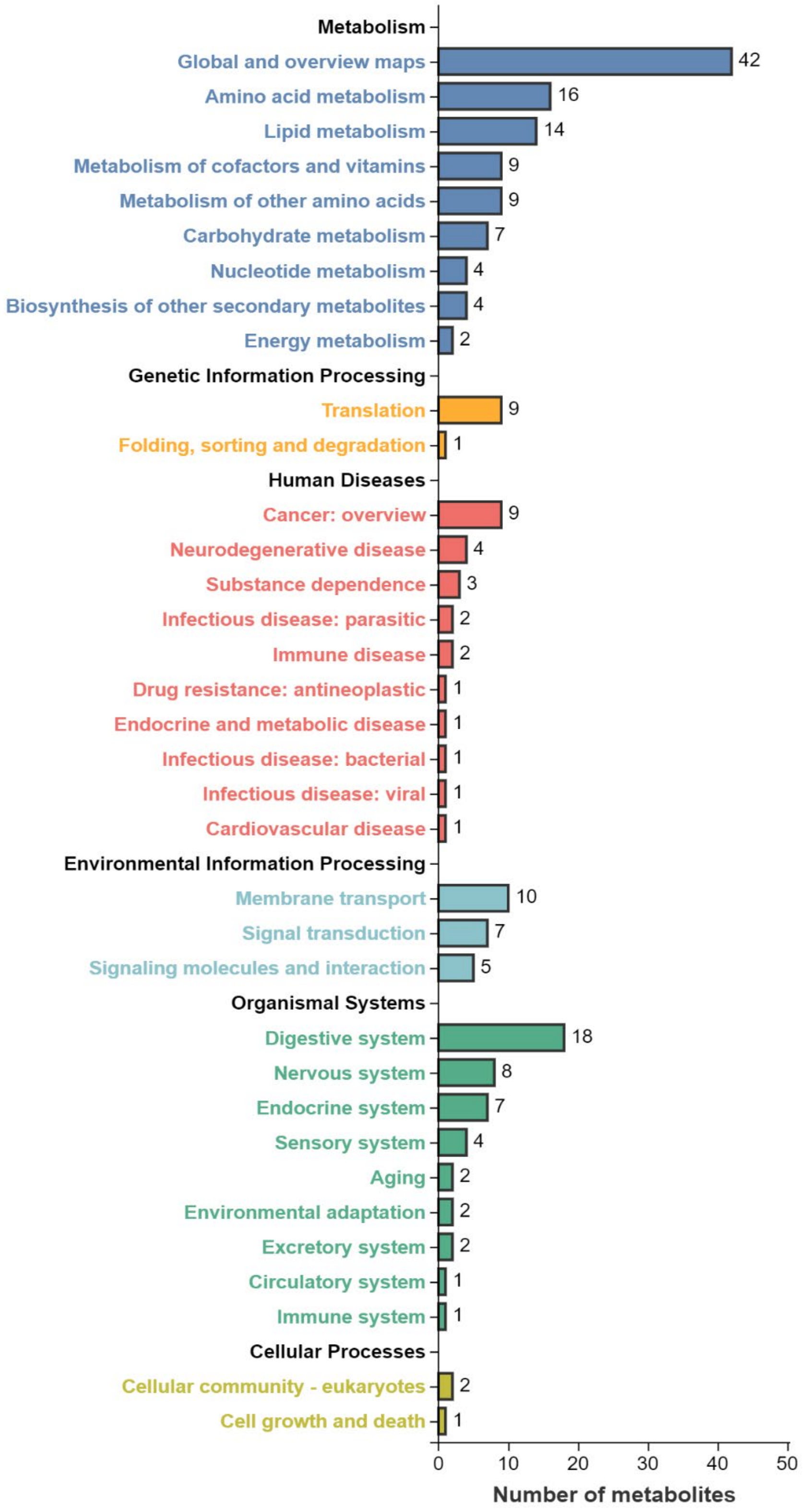

Supplementary Figure 8. KEGG annotation result of identified metabolites from single MCF-7 cells in positive mode. Identified metabolites mainly relates to fundamental energy metabolism, signal transduction, aging and human diseases.

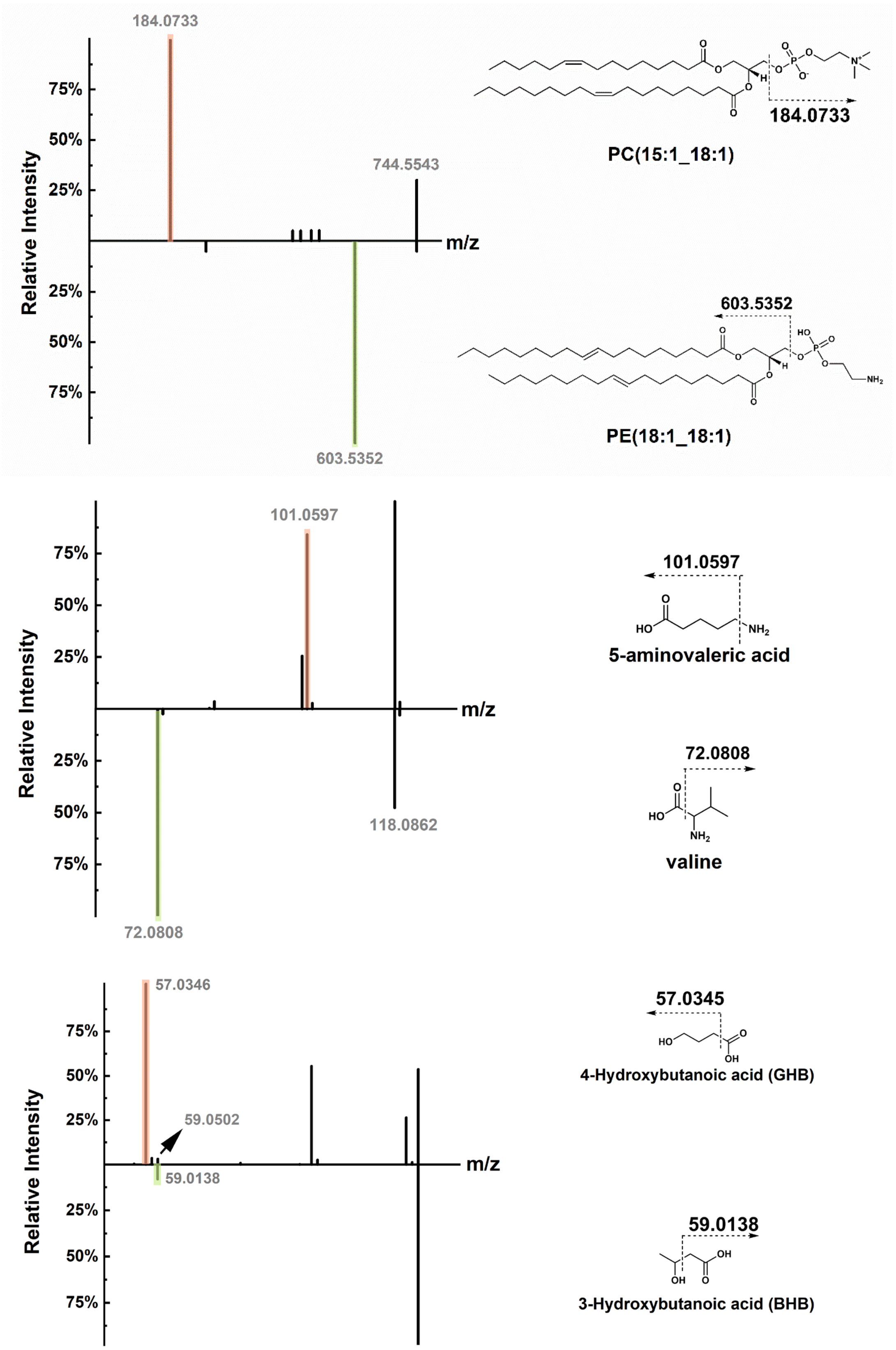

Supplementary Figure 9. Reference MS^2 spectra of PC(15:1_18:1) and PE(18:1_18:1) (*m/z* 744.5543), 5-aminovaleric acid and valine (*m/z* 118.0862) and 4-hydroxybutanoic acid and 3-hydroxybutanoic acid (*m/z* 103.0401). Characteristic ion pairs were highlighted with different color and the proposed fragmentation position was indicated.

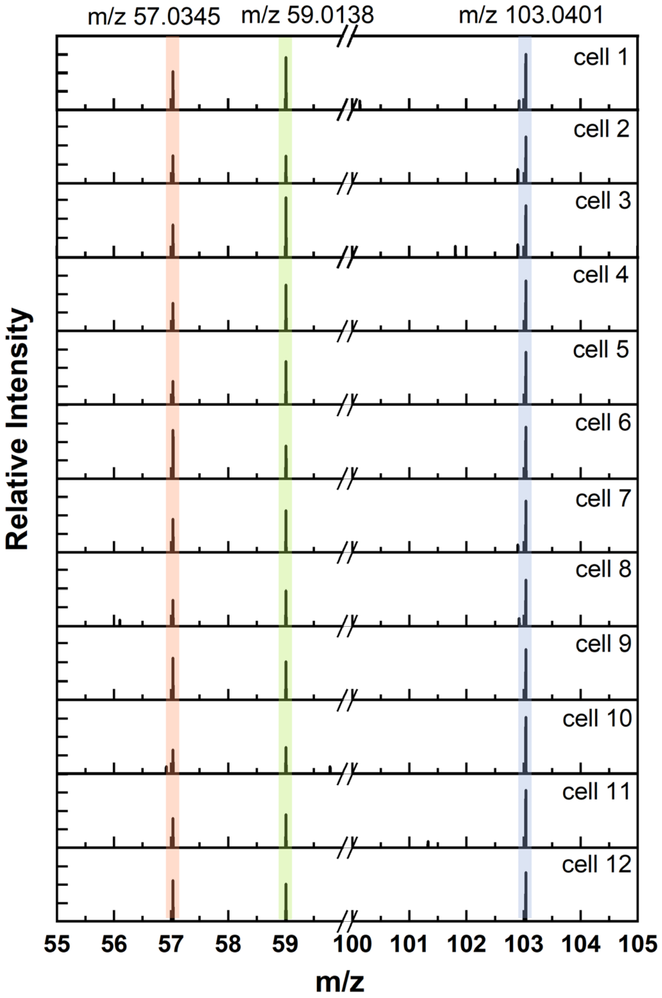

Supplementary Figure 10. MS^2 spectrum of ions at *m/z* 103.0401 among random 12 cells in ID-organic cytoMS. Parent ion (*m/z* 103,0401) and characteristic fragment ions (*m/z* 57.0345 and *m/z* 59.0138) were highlighted with different color.

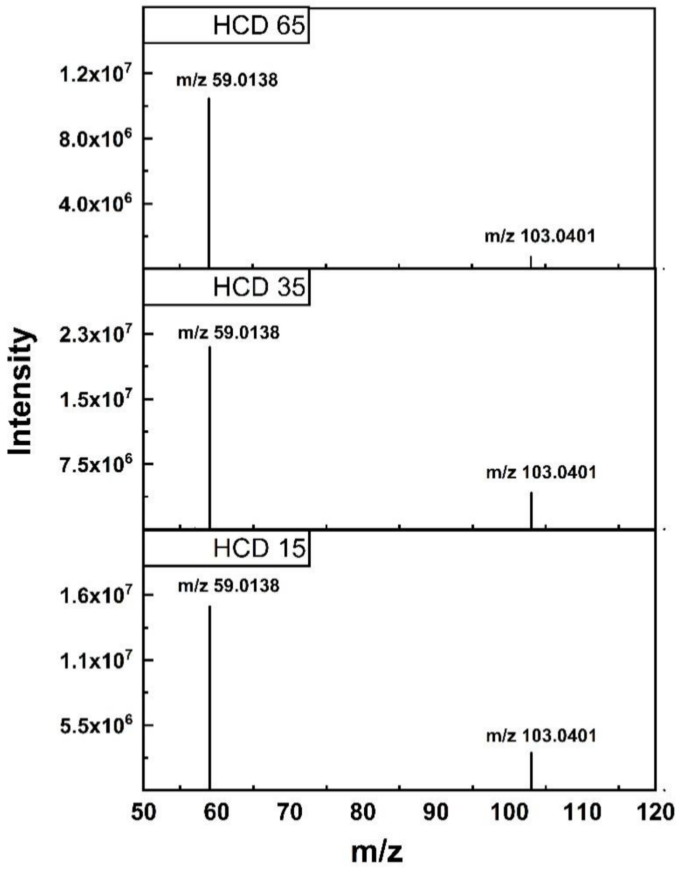

Supplementary Figure 11. Standard MS^2 spectra of 3-hydroxybutanoic acid (BHB) with different HCD collision energy. No mass peak of *m/z* 57.0345 appeared at all collision energy.

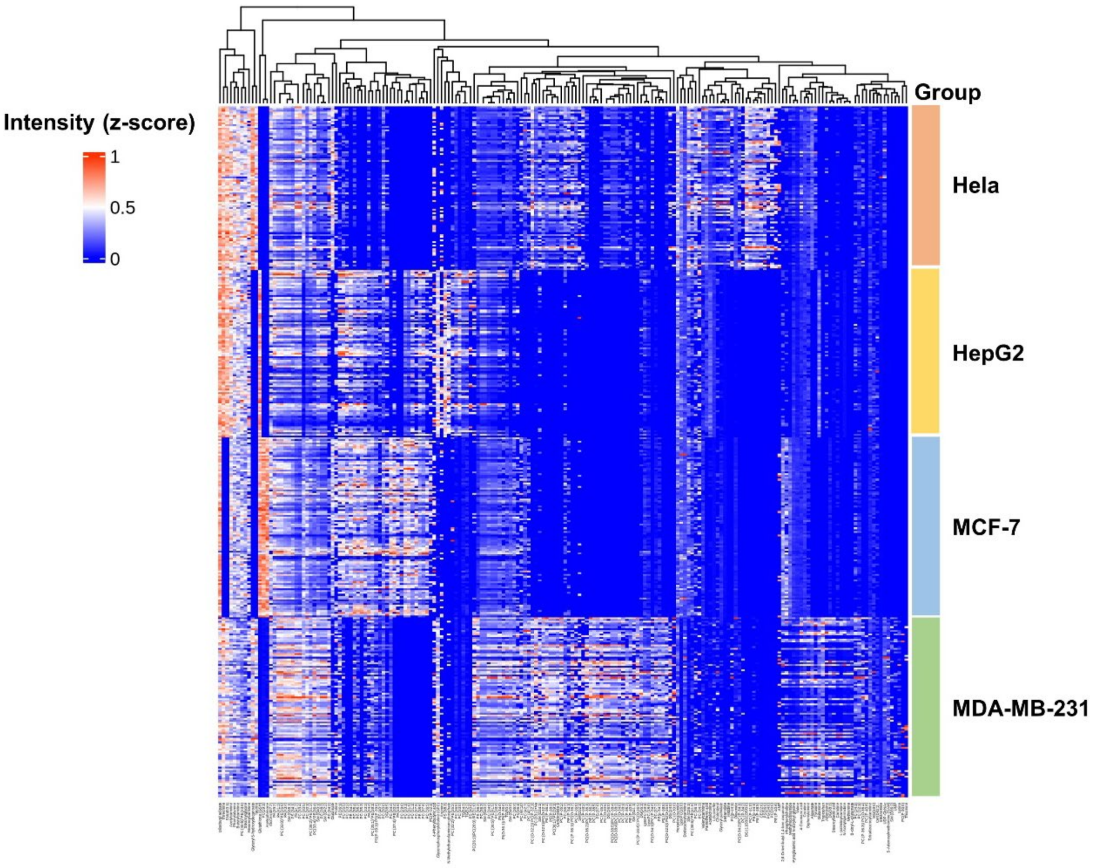

Supplementary Figure 12. Heat maps of the z-score normalized intensity of the single cell metabolites in four tumor cell types. (Breast cancer cell: MCF-7, MDA-MB-231. Cervical cancer cell: Hela. Hepatocellular cancer cell: HepG2). These cell types exhibit distinctive metabolic pattern.

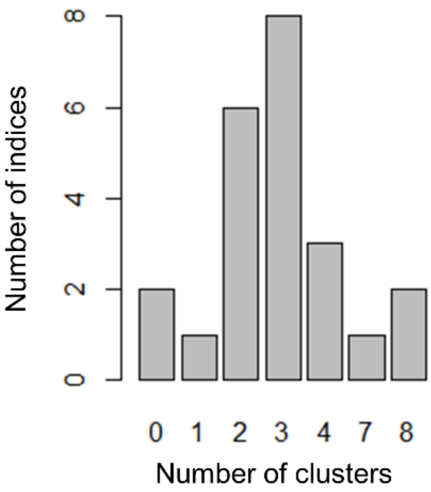

Supplementary Figure 13. Cluster number determination with metabolome data (with BHB/GHB) isomer using K-means clustering method with the Nbclust package in R. The clustering number with the highest support for the indicators (y axis) will be selected[1].

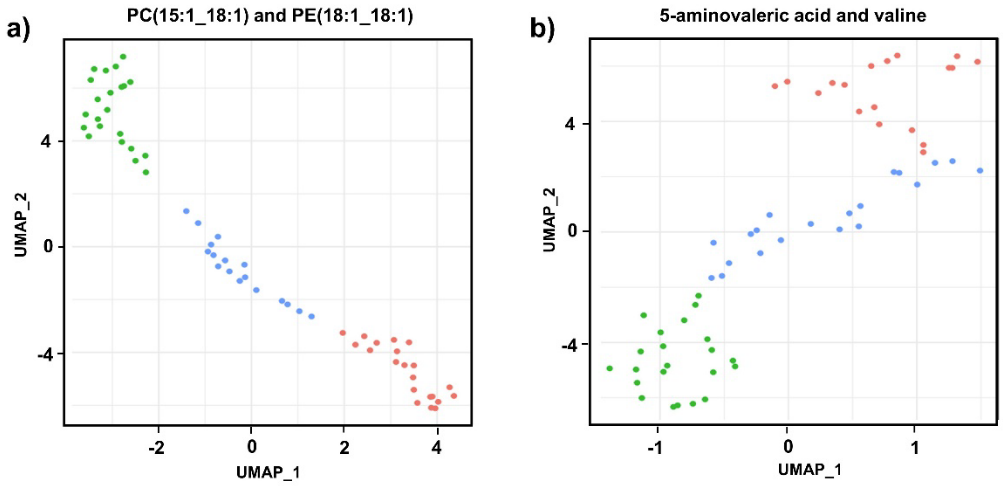

Supplementary Figure 14. UMAP visualization results of MCF-7 cell metabolome data with isomer information of **a**. PC(15:1_18:1) and PE(18:1_18:1) and **b**. 5-aminovaleric acid and valine. K-means clustering method was utilized to cluster cells into three clusters, which annotated with different color.

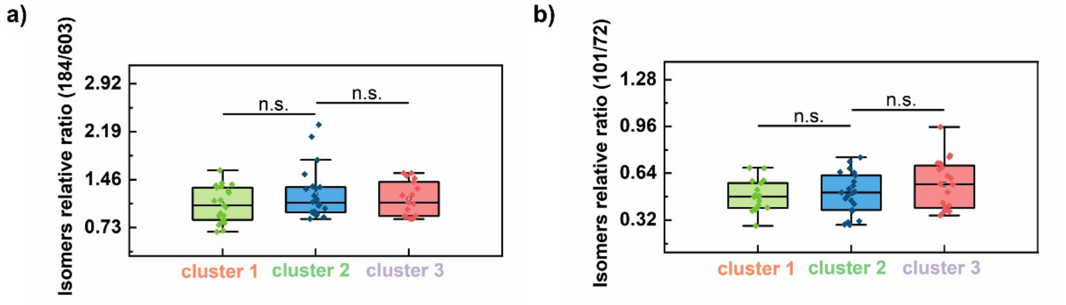

Supplementary Figure 15. a PC (15:1_18:1) and PE (18:1_18:1) isomers relative abundance (MS peak intensity of m/z 184.0733 vs m/z 603.5352) among above 3 clusters **b** 5-aminovaleric acid and valine isomers relative abundance (MS peak intensity of m/z 101.0597 vs m/z 72.0808) among above 3 clusters (n=60 cells in total. Boxplot shows median, 0.25 and 0.75 quantile, and whiskers extend to points within 1.5 interquartile range of lower and upper quartile. Kruskal-Wallis test)

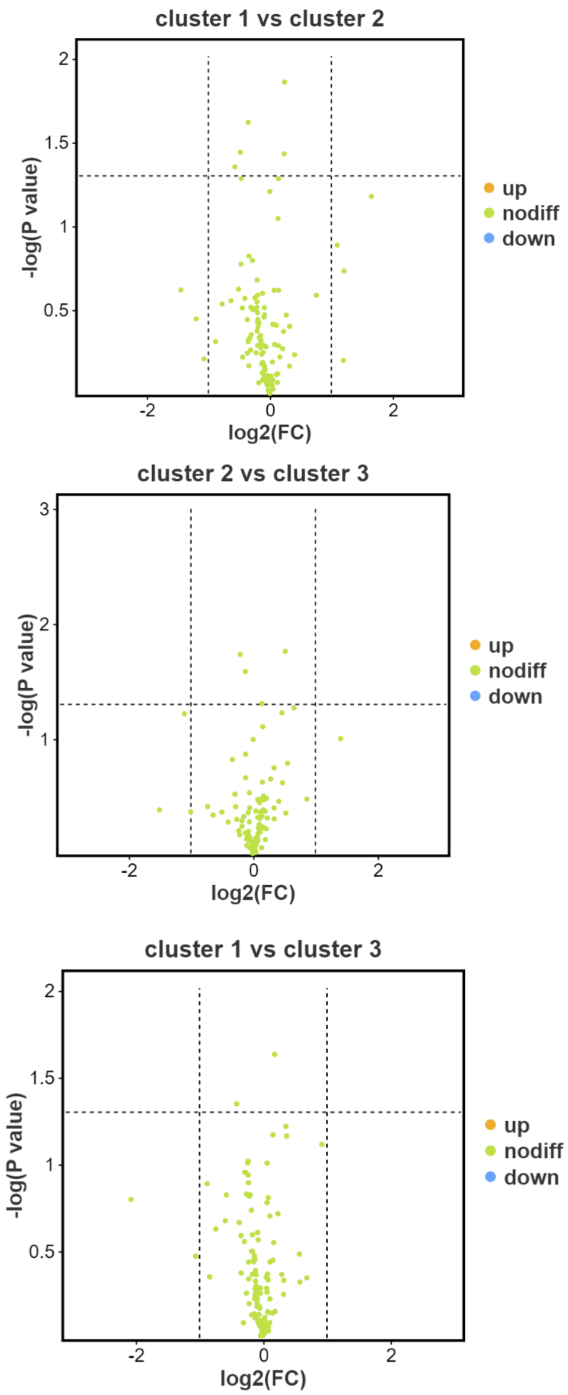

Supplementary Figure 16. Volcano plot of the P-values and fold changes (FC) of normalized intensities for metabolite signals between cluster 1, 2 and 3; (T test, P value <0.05, -log10 transformation was used for P values; ∣ FC ∣ >2). Dashed horizontal line marks the significance threshold of 0.05 and dashed vertical lines mark the effect size threshold of an absolute log2 fold change of 1. Dots represent identified metabolites, with up-regulated metabolites marked as orange and down-regulated metabolites marked as blue. No significant metabolites abundance difference occurred among clusters.

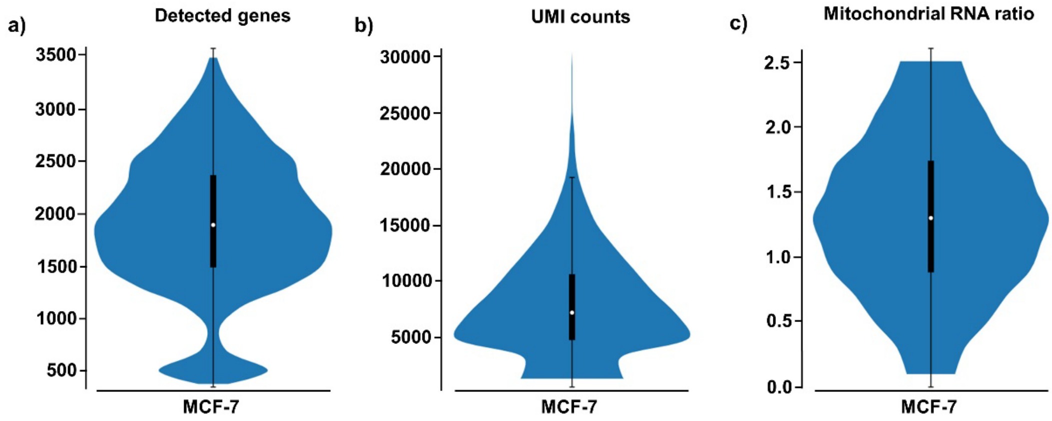

Supplementary Figure 17. (a) Violin plot of mRNA expression among cells (b) Violin plot of mRNA read counts among cells (c) Violin plot of mitochondrial RNA ratio among cells.

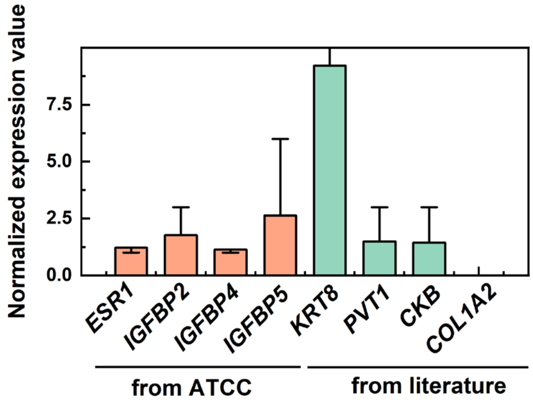

Supplementary Figure 18. Bar plot of expression level of MCF-7 cell markers which derived from ATCC database. Expression changes of latter specific four genes also correspond with reported conclusion[2-6] in MCF-7 cells. (Barplots shows median, 0.25 and 0.75 quantile, and whiskers extend to points within 1.5 interquartile range of lower and upper quartile, n=10373 cells in total

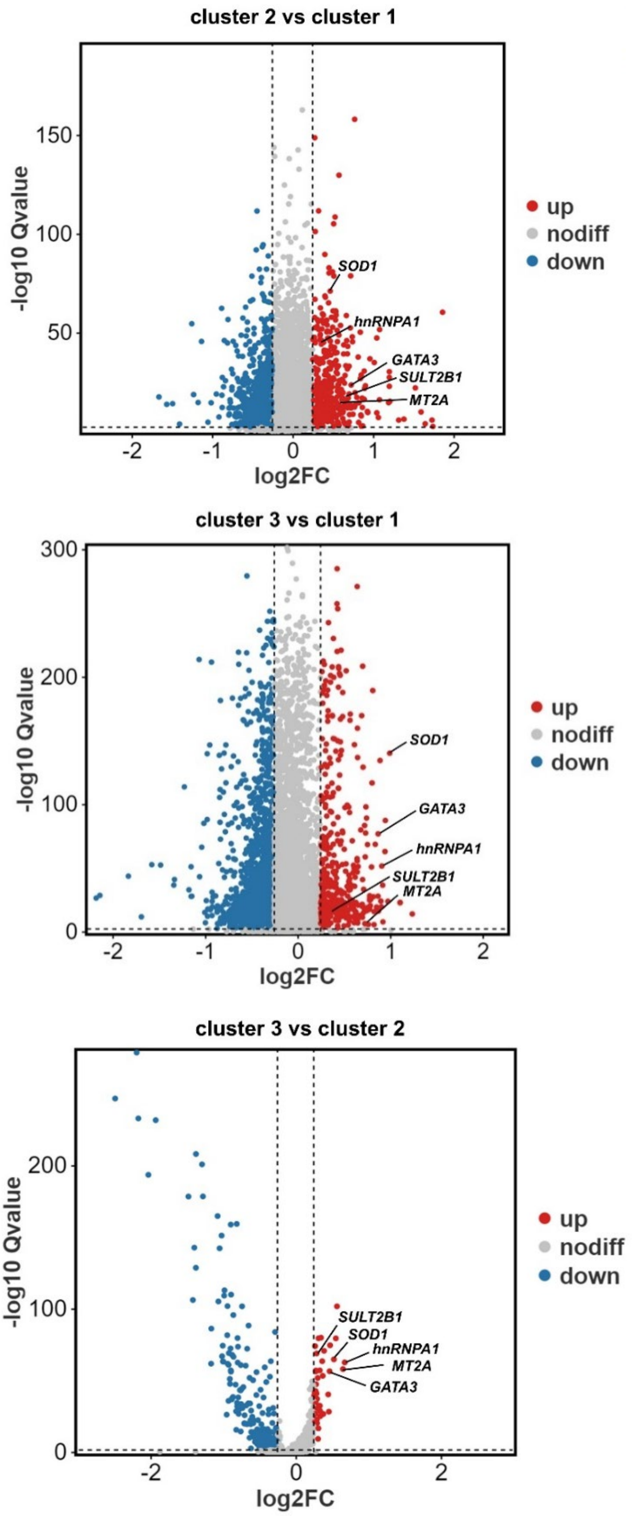

Supplementary Figure 19. Volcano plot of differential genes expression analysis among 3 clusters from single cell sequencing data. (n=10373 cells in total, dots represent differential genes, with up-regulated genes marked as red and down-regulated genes marked as blue. BHB downstream target proteins were annotated (wilcoxon test, log2|FC| >= 0.25, Q values <= 0.05)

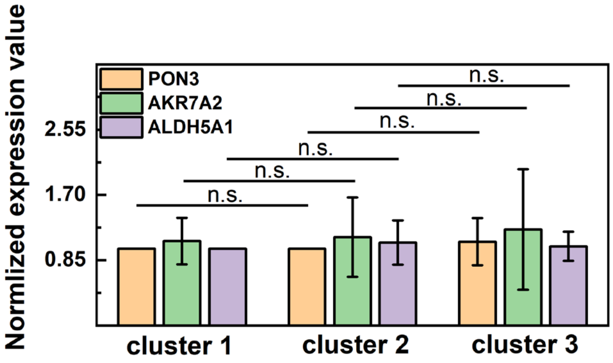

Supplementary Figure 20. Bar plot of normalized expression level of *PON3, AKR7A2* and *ALDH5A1* among 3 cell clusters. (Barplots shows mean, error bar represents the standard deviation, n=10373 cells in total, Kruskal-Wallis test)

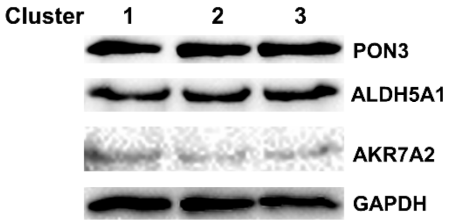

Supplementary Figure 21. WB results of PON3 (Lactonase 3), ALDH5A1 (aldehyde dehydrogenase 5 family member A1) and AKR7A2 (aldo-keto reductase family 7 member A2) among 3 clusters.

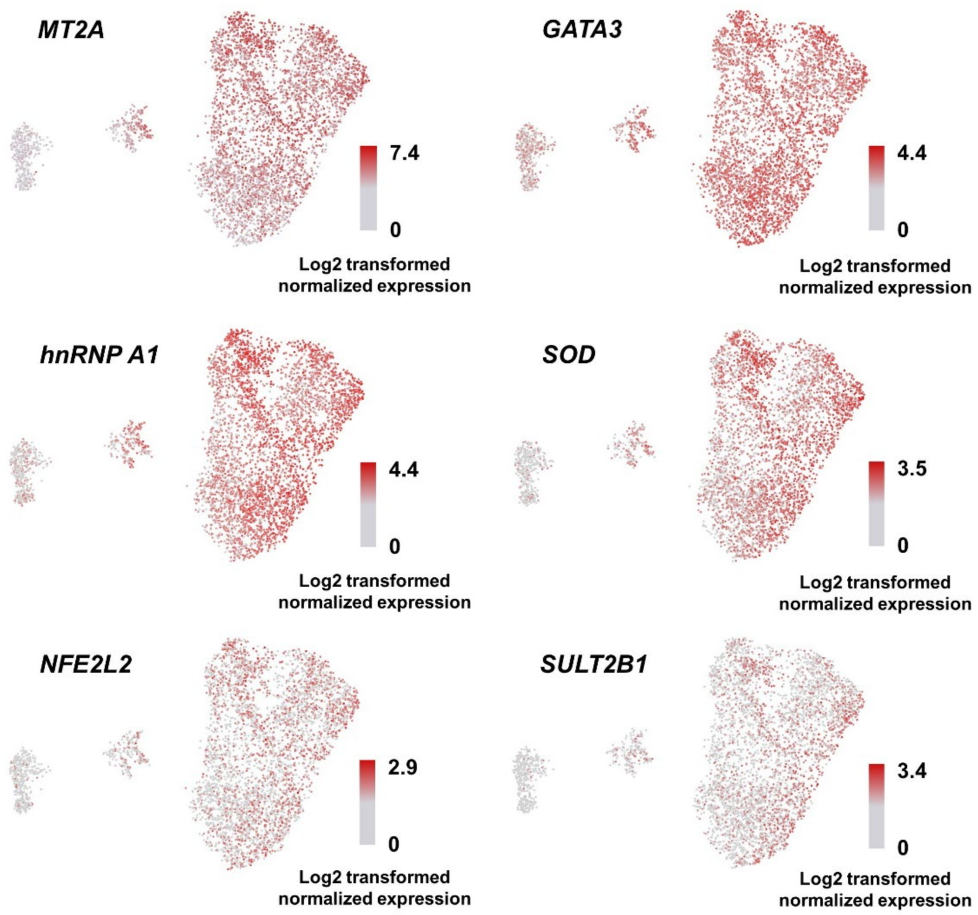

Supplementary Figure 22. UMAP plot of genes encoding BHB downstream target proteins within MCF-7 cells

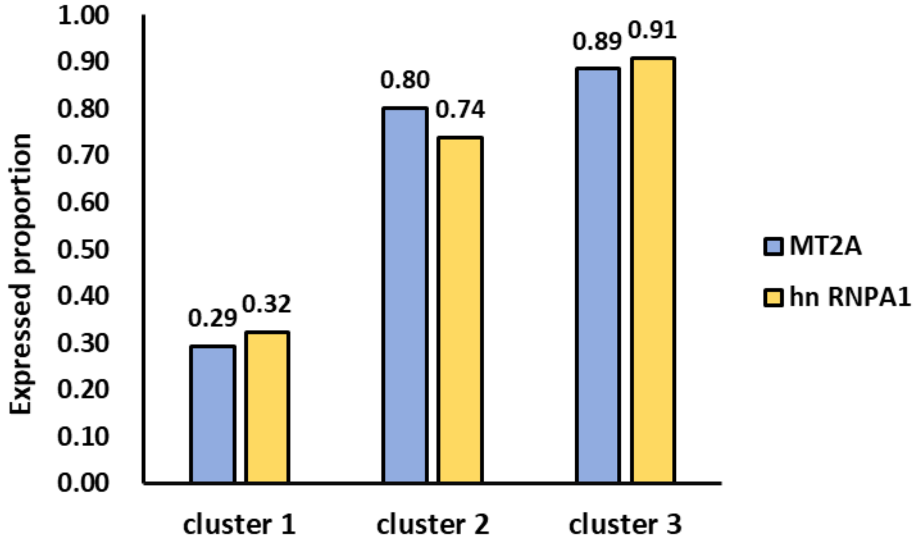

Supplementary Figure 23. Expressed cell proportion of *MT2A* and *hnRNP A1* among clusters.

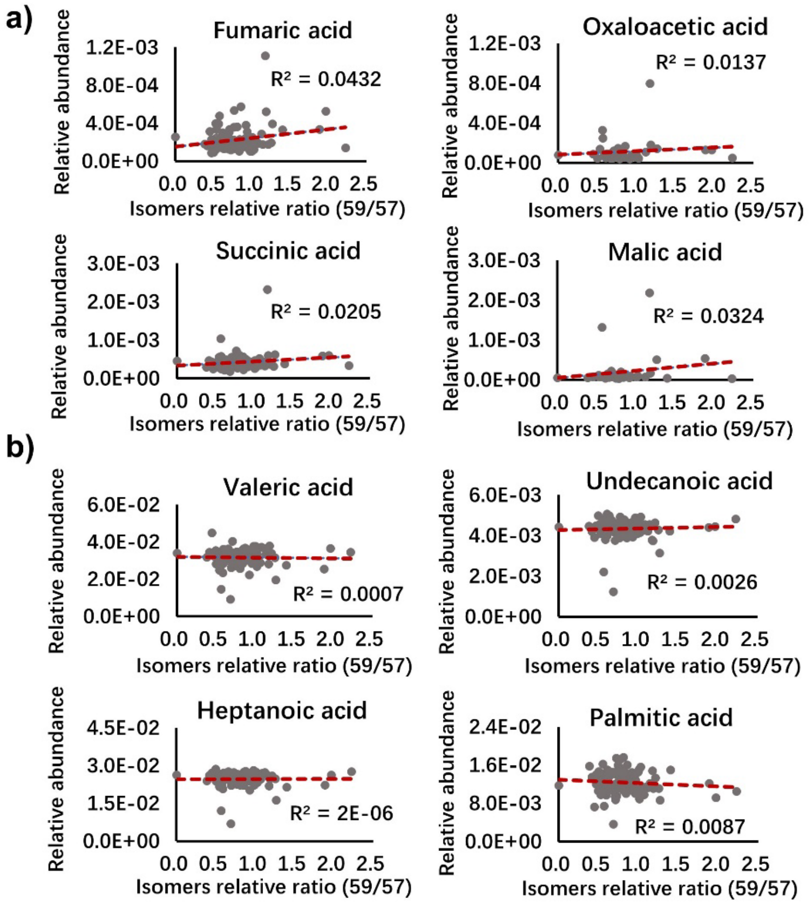

Supplementary Figure 24. Scatter plot of relative abundance of metabolites related to TCA cycle and fatty acids metabolism with BHB/GHB isomers relative ratio. (a) TCA cycle. (b) Fatty acids metabolism.

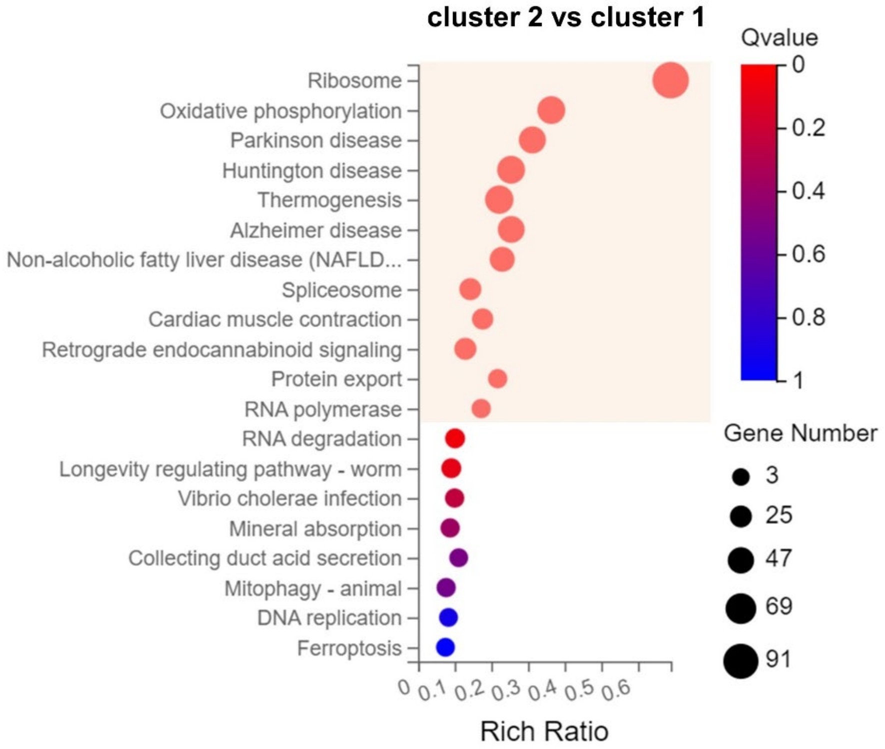
cluster 2 vs cluster 1
Ribosome
Oxidative phosphorylation
Parkinson disease
Huntington disease
Thermogenesis
Alzheimer disease
Non-alcoholic fatty liver disease (NAFLD...
Spliceosome
Cardiac muscle contraction
Retrograde endocannabinoid signaling
Protein export
RNA polymerase
RNA degradation
Longevity regulating pathway - worm
Vibrio cholerae infection
Mineral absorption
Collecting duct acid secretion
Mitophagy - animal
DNA replication
Ferroptosis
0 0.1 0.2 0.3 0.4 0.5 0.6
Rich Ratio
Qvalue
0 0.2 0.4 0.6 0.8 1
Gene Number
3 25 47 69 91

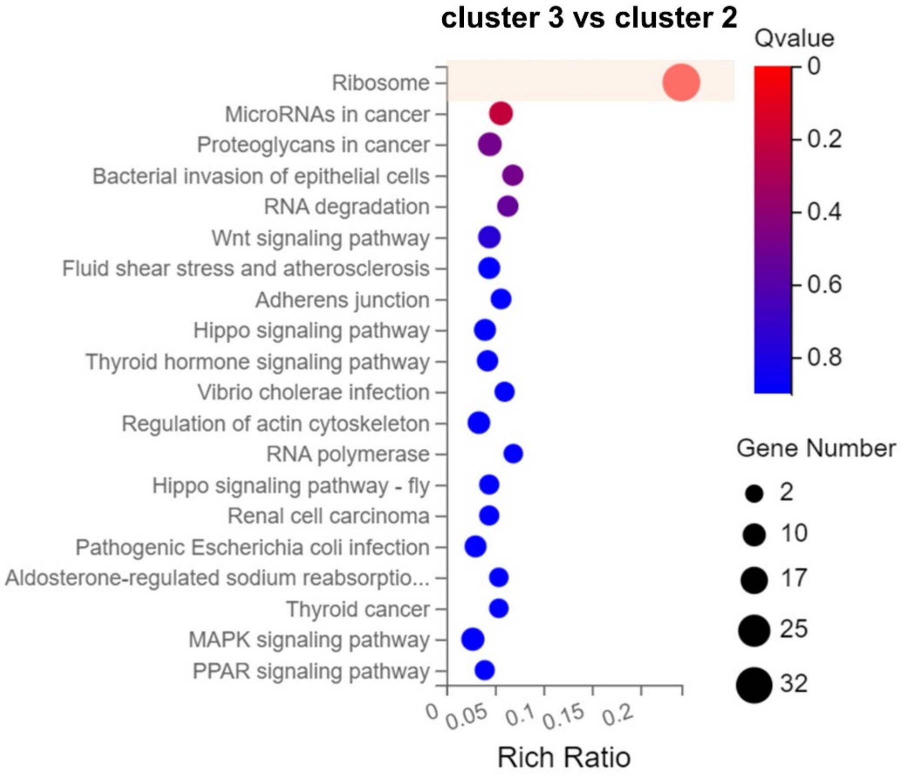
cluster 3 vs cluster 2
Ribosome
MicroRNAs in cancer
Proteoglycans in cancer
Bacterial invasion of epithelial cells
RNA degradation
Wnt signaling pathway
Fluid shear stress and atherosclerosis
Adherens junction
Hippo signaling pathway
Thyroid hormone signaling pathway
Vibrio cholerae infection
Regulation of actin cytoskeleton
RNA polymerase
Hippo signaling pathway - fly
Renal cell carcinoma
Pathogenic Escherichia coli infection
Aldosterone-regulated sodium reabsorptio...
Thyroid cancer
MAPK signaling pathway
PPAR signaling pathway
0 0.05 0.1 0.15 0.2
Rich Ratio
Qvalue
0 0.2 0.4 0.6 0.8
Gene Number
2 10 17 25 32

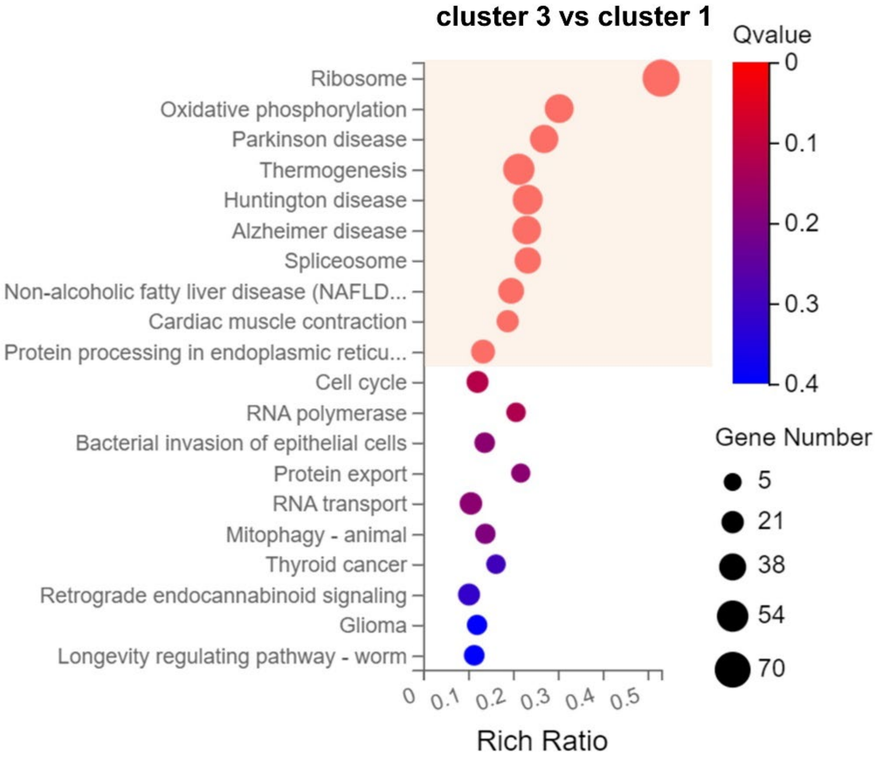

Supplementary Figure 25. KEGG pathway enrichment bubble diagrams among 3 clusters. phyper function in R software was used to perform the enrichment analysis and calculate the P-values, which further corrected to Q-values with BH method. Significant enriched pathways were highlighted (Q value <= 0.05). RNA degradation and neuron degenerative diseases related genes were significantly rich.

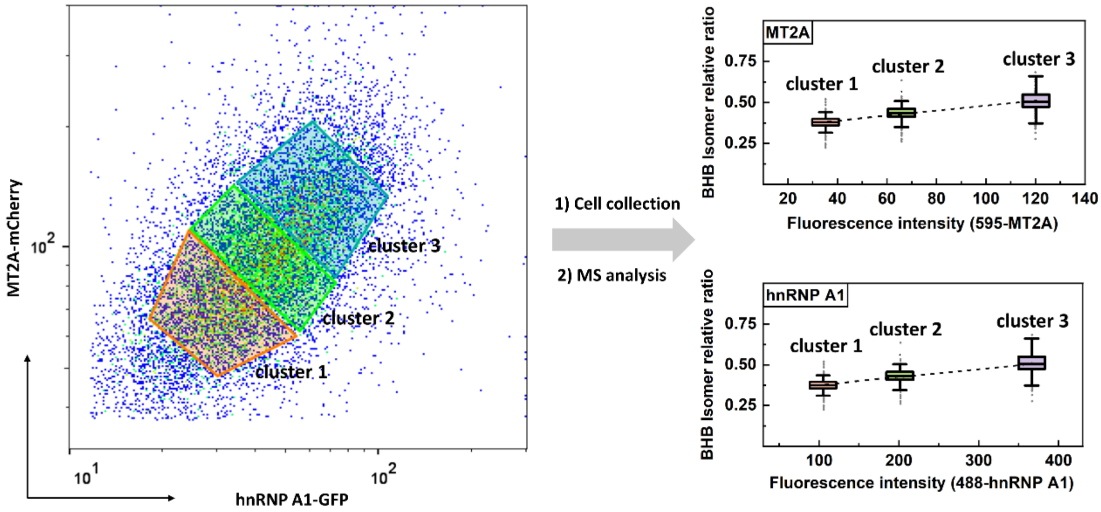

Supplementary Figure 26. Based on fluorescence intensity of MT2A-mCherry and hnRNP A1-GFP, MCF-7 cells were fractioned into 3 clusters. MS analysis was conducted to quantify BHB isomer relative ratio of above clusters. (Figure 6c-d)

Supplementary Table 1. Quality control result of single cell RNA sequencing

Median Genes per Cell	Total Genes Detected	Median UMI Counts per Cell
1897	23549	7072

References:

1. Charrad, M., Ghazzali, N., Boiteau, V., Niknafs, A., NbClust: An R Package for Determining the Relevant Number of Clusters in a Data Set, *Journal of Statistical Software* **61**, 1-36 (2014).
2. Isakova, A., Neff, N., Quake, S. R., Single-cell quantification of a broad RNA spectrum reveals unique noncoding patterns associated with cell types and states, *Proc. Natl. Acad. Sci. U. S. A.* **118**, e2113568118 (2021).
3. Qiu, J., Du, Z., Wang, Y., Zhou, Y., Zhang, Y., Xie, Y., Lv, Q., Weighted gene co-expression network analysis reveals modules and hub genes associated with the development of breast cancer, *Medicine* **98**, (2019).
4. Zhou, C., Nitschke, A. M., Xiong, W., Zhang, Q., Tang, Y., Bloch, M., Elliott, S., Zhu, Y., Bazzone, L., Yu, D., Weldon, C. B., Schiff, R., McLachlan, J. A., Beckman, B. S., Wiese, T. E., Nephew, K. P., Shan, B., Burow, M. E., Wang, G., Proteomic analysis of tumor necrosis factor-α resistant human breast cancer cells reveals a MEK5/Erk5-mediated epithelial-mesenchymal transition phenotype, *Breast Cancer Research* **10**, (2008).
5. Foulstone, E. J., Zeng, L., Perks, C. M., Holly, J. M. P., Insulin-Like Growth Factor Binding Protein 2 (IGFBP-2) Promotes Growth and Survival of Breast Epithelial Cells: Novel Regulation of the Estrogen Receptor, *Endocrinology* **154**, 1780-1793 (2013).
6. Montes-de-Oca-Fuentes, E. V., Jacome-Lopez, K., Zarco-Mendoza, A., Guerrero, G., Ventura-Gallegos, J. L., Juarez-Mendez, S., Cabrera-Quintero, A. J., Recillas-Targa, F., Zentella-Dehesa, A., Differential DNA methylation and CTCF binding between the ESR1 promoter a of MCF-7 and MDA-MB-231 breast cancer cells, *Mol. Biol. Rep.* **51**, (2024).
