# Supplementary material for: In-depth organic mass cytometry reveals differential contents of 3-hydroxybutanoic acid at the single-cell level
Source: Nat Commun. 2024 May 23;15:4387. doi: 10.1038/s41467-024-48865-2 (PMC11116506; doi:10.1038/s41467-024-48865-2)
Supplement: Supplementary file 3 — Reporting Summary [file 41467_2024_48865_MOESM3_ESM.pdf]

nature portfolio

Corresponding author(s): Yu Bai DAPR NCOMMS-23-27831

Last updated by author(s): May 9, 2024

Reporting Summary

Nature Portfolio wishes to improve the reproducibility of the work that we publish. This form provides structure for consistency and transparency in reporting. For further information on Nature Portfolio policies, see our Editorial Policies and the Editorial Policy Checklist.

Statistics

For all statistical analyses, confirm that the following items are present in the figure legend, table legend, main text, or Methods section.

n/a	Confirmed	
☐	☒	The exact sample size (*n*) for each experimental group/condition, given as a discrete number and unit of measurement
☐	☒	A statement on whether measurements were taken from distinct samples or whether the same sample was measured repeatedly
☐	☒	The statistical test(s) used AND whether they are one- or two-sided<br>*Only common tests should be described solely by name; describe more complex techniques in the Methods section.*
☐	☒	A description of all covariates tested
☐	☒	A description of any assumptions or corrections, such as tests of normality and adjustment for multiple comparisons
☐	☒	A full description of the statistical parameters including central tendency (e.g. means) or other basic estimates (e.g. regression coefficient) AND variation (e.g. standard deviation) or associated estimates of uncertainty (e.g. confidence intervals)
☐	☒	For null hypothesis testing, the test statistic (e.g. *F*, *t*, *r*) with confidence intervals, effect sizes, degrees of freedom and *P* value noted<br>*Give P values as exact values whenever suitable.*
☒	☐	For Bayesian analysis, information on the choice of priors and Markov chain Monte Carlo settings
☐	☒	For hierarchical and complex designs, identification of the appropriate level for tests and full reporting of outcomes
☒	☐	Estimates of effect sizes (e.g. Cohen's *d*, Pearson's *r*), indicating how they were calculated

Our web collection on statistics for biologists contains articles on many of the points above.

Software and code

Policy information about availability of computer code

Data collection	Xcalibur 4.0 from Thermo Scientific
Data analysis	Xcalibur 4.0 from Thermo Scientific, Compound Discover 3.3, Dr. Tom Multi-Omics Data Mining System, Nbclust package in R, Lipidsearch 5.1

For manuscripts utilizing custom algorithms or software that are central to the research but not yet described in published literature, software must be made available to editors and reviewers. We strongly encourage code deposition in a community repository (e.g. GitHub). See the Nature Portfolio guidelines for submitting code & software for further information.

Data

Policy information about availability of data

All manuscripts must include a data availability statement. This statement should provide the following information, where applicable:

- Accession codes, unique identifiers, or web links for publicly available datasets
- A description of any restrictions on data availability
- For clinical datasets or third party data, please ensure that the statement adheres to our policy

Single cell sequencing data that support the findings of this study have been deposited in Gene Expression Omnibus (GEO) with the accession codes GSE262591 at https://www.ncbi.nlm.nih.gov/geo/query/acc.cgi?acc=GSE262591. The metabolomic MS raw data have been deposited to MetaboLights with the dataset identifier MTBLS8113. The remaining data are available within the article, Supplementary Information or Source Data file. Source data are provided with this paper.

Research involving human participants, their data, or biological material

Policy information about studies with human participants or human data. See also policy information about sex, gender (identity/presentation), and sexual orientation and race, ethnicity and racism.

Reporting on sex and gender	n/a
Reporting on race, ethnicity, or other socially relevant groupings	n/a
Population characteristics	n/a
Recruitment	n/a
Ethics oversight	n/a

Note that full information on the approval of the study protocol must also be provided in the manuscript.

Field-specific reporting

Please select the one below that is the best fit for your research. If you are not sure, read the appropriate sections before making your selection.

- [x] Life sciences
- [ ] Behavioural & social sciences
- [ ] Ecological, evolutionary & environmental sciences

For a reference copy of the document with all sections, see nature.com/documents/nr-reporting-summary-flat.pdf

Life sciences study design

All studies must disclose on these points even when the disclosure is negative.

Sample size	Metabolomics data from 138 MCF-7 cells were utilized for metabolites identification and KEGG analysis. 100 MCF-7 cells, 91 Hela cells, 100 MDA-MB-231 cells and 93 HepG2 cells were used for cell types discrimination. Single cell sequencing was performed using 10373 MCF-7 cells. 208 MCF-7 cells were utilized for FACS sorting verification. No sample-size calculation was performed for single cell metabolomics because these samples were just used to discriminate different cell types and clusters, not for actual biomarker discovery. Cell numbers for sequencing is set to meet statistics analysis requirement of 10X genomics.
Data exclusions	Metabolites that are not expressed in more than 30% of all cells will be excluded from statistics analysis.
Replication	For BHB/GHB isomer discrimination assay, 3 independent experiments were performed with identical results.<br>For extension analysis performance validation in single cells, at least 5 independent experiments were performed with identical results.
Randomization	MCF-7 cells were unsupervisedly clustered into 3 subtypes. No further experimental group was applied.
Blinding	Blinding was not applied because cell types information was required.

Reporting for specific materials, systems and methods

We require information from authors about some types of materials, experimental systems and methods used in many studies. Here, indicate whether each material, system or method listed is relevant to your study. If you are not sure if a list item applies to your research, read the appropriate section before selecting a response.

Materials & experimental systems

n/a	Involved in the study	
	x	Antibodies
	x	Eukaryotic cell lines
x		Palaeontology and archaeology
x		Animals and other organisms
x		Clinical data
x		Dual use research of concern
x		Plants

Methods

n/a	Involved in the study	
x		ChIP-seq
	x	Flow cytometry
x		MRI-based neuroimaging

Antibodies

Antibodies used	Anti-Metallothionein antibody (Mouse monoclonal, 1/1000 dilution used, abcam, ab12228, UC1MT, GR3415395-4) , Recombinant Anti-hnRNP A1 antibody (Rabbit monoclonal, 1/1000 dilution used, abcam, ab177152, EPR12768, GR139101-6), Recombinant

ALDH5A1/SSADH antibody (Rabbit monoclonal, 1/10000 dilution used, abcam, ab129017, EPR7794, GR3440323-7), anti AKR7A2 antibody (Rabbit polyclonal, 1/500 dilution used, abcam, ab97458, GR73247-6), anti PON3 antibody (Rabbit monoclonal, 1/1000 dilution used, abcam, ab109258, EPR2903(2), 1068361-1). HRP-conjugated GAPDH Monoclonal antibody (Mouse monoclonal, 1/3000 dilution used, proteintech, HRP-60004, 1E6D9, 21005148). Secondary antibody (green) Alexa Fluor® 488 donkey anti-rabbit IgG (H+L) (Donkey polyclonal, used at a 1/3000 dilution, invitrogen, R37118, 2376850), Secondary antibody (red) Alexa Fluor® 594 donkey anti-mouse IgG (H+L) (Donkey polyclonal, used at a 1/3000 dilution, invitrogen, R37115, 2474956). All antibodies above were commercially purchased.

Validation	Promise guarantee from abcam covers the use of ab12228 and ab177152 in the flow cytometry, ab129017, ab97458 and ab109258 in the WB assay.

Eukaryotic cell lines

Policy information about cell lines and Sex and Gender in Research

Cell line source(s)	MCF-7 were purchased from Union Cell Resource Center (Beijing, China), MDA-MB-231, Hela and HepG2 were kind gifts from Xinxiang Zhang Laboratory (Peking University),
Authentication	Cell lines authenticated by short tandem repeat (STR) testing
Mycoplasma contamination	The cell lines were not tested for mycoplasma contamination.
Commonly misidentified lines (See ICLAC register)	No commonly misidentified cell lines used.

Plants

Seed stocks	N/A
Novel plant genotypes	N/A
Authentication	N/A

Flow Cytometry

Plots

Confirm that:

- [x] The axis labels state the marker and fluorochrome used (e.g. CD4-FITC).
- [x] The axis scales are clearly visible. Include numbers along axes only for bottom left plot of group (a 'group' is an analysis of identical markers).
- [ ] All plots are contour plots with outliers or pseudocolor plots.
- [ ] A numerical value for number of cells or percentage (with statistics) is provided.

Methodology

Sample preparation	Sample pretreatment method refers to provided Indirect flow cytometry protocol from abcam official website. Briefly, harvest cells (~10e7 cells/tube) were fixed by 4% paraformaldehyde solution, followed by permeabilization by 0.5% Triton X-100 in PBS. Primary and fluorochrome-labeled secondary antibody working solution, which both diluted to 1 μg/ml in 3% BSA in PBS, was added one after another for target protein labeling. After cell washing with PBS, cells were hold at 4 °C and analyzed as soon as possible
Instrument	Astrios EQ (BeckMan Coulter, USA)
Software	FlowJo_V10
Cell population abundance	Based on fluorescence intensity of MT2A-mCherry and hnRNP A1-GFP, MCF-7 cells were fractioned into 3 clusters.
Gating strategy	FSC/SSC, MT2A-mCherry/hnRNP A1-GFP

- [x] Tick this box to confirm that a figure exemplifying the gating strategy is provided in the Supplementary Information.
